# Premorbid of depressive youth at clinical high-risk for psychosis

**DOI:** 10.1192/j.eurpsy.2022.1757

**Published:** 2022-09-01

**Authors:** M. Omelchenko

**Affiliations:** Mental Health Research Centre, Youth Psychiatry, Moscow, Russian Federation

**Keywords:** Youth depression, prevention, Clinical high-risk, premorbid

## Abstract

**Introduction:**

Early detection of psychosis is a promising area in preventive psychiatry. The use of early intervention can prevent the first episode psychosis and improve outcomes.

**Objectives:**

Identification of premorbid features of depressive patients at clinical high risk for psychosis (CHR) comparing with depressive patients without CHR in order to improve early recognition of the psychotic process.

**Methods:**

219 young depressive in-patients with CHR criteria for SOPS with attenuated positive and attenuated negative symptoms and 52 young depressive in-patients without CHR were examined. Presence of obstetric complications, neurodevelopmental deviance, neurological and psychiatric signs at the premorbid stage, and the level of premorbid functioning on the PAS were examined.

**Results:**

It has been established that depressive patients at CHR and without CHR had some obstetric complications (57.5% and 40.4%, respectively). Neurodevelopmental deviance in the first year of live was in 57.5% patients with CHR. At the age of 3-5 sleep disorders, ADHD and phobias were more common in patients at CHR than without it (58.8% and 32.7%, p=0.014). In pubertal, patients at CHR were more likely to show depression symptoms, obsessions, and aggression - 90.4% versus 76.9% (p=0.029). On the PAS scale, a decrease of the level of premorbid functioning has been observed in two groups of patients with and without CHR from the age of 12: from 12 to 15 years, 0.4 and 0.3 (p=0.004), from 16 to 18 years, 0.47 and 0.37 (p 0.001).

**Conclusions:**

Premorbid functioning were worst in patients with CHR, which indicates the possibility of early clinical detection of psychosis.

**Disclosure:**

No significant relationships.

